# Supplementary material for: Heat adaptation of phage T7 under an extended genetic code
Source: Virus Evol. 2021 Dec 1;7(2):veab100. doi: 10.1093/ve/veab100 (PMC8923235; doi:10.1093/ve/veab100)
Supplement: veab100_Supp [file veab100_supp.zip › Sequencing_Analysis.zip!/Sequencing_Analysis/S2/calibration/0.unique_coverage.pdf]

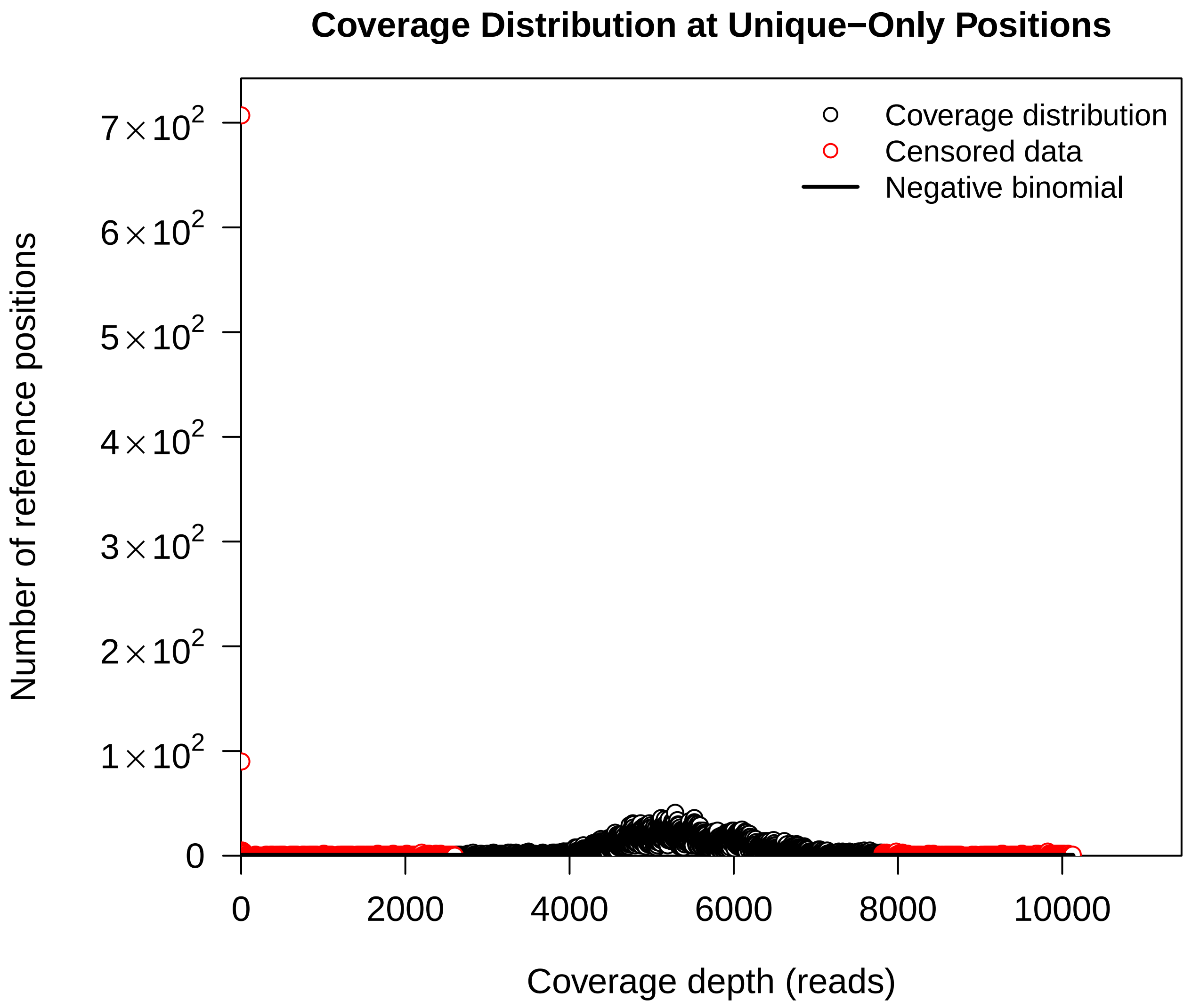

Coverage Distribution at Unique−Only Positions
Coverage distribution
Censored data
Negative binomial
Number of reference positions
0
1×10^2
2×10^2
3×10^2
4×10^2
5×10^2
6×10^2
7×10^2
0
2000
4000
6000
8000
10000
Coverage depth (reads)
